# X-ray-Fluorescence Imaging for In Vivo Detection of Gold-Nanoparticle-Labeled Immune Cells: A GEANT4 Based Feasibility Study

**DOI:** 10.3390/cancers13225759

**Published:** 2021-11-17

**Authors:** Arthur Ungerer, Theresa Staufer, Oliver Schmutzler, Christian Körnig, Kai Rothkamm, Florian Grüner

**Affiliations:** 1 University Medical Center Hamburg-Eppendorf, Department of Radiotherapy and Radiation Oncology, Medical Faculty, University of Hamburg, Martinistraße 52, 20246 Hamburg, Germany; arthur.ungerer@stud.uke.uni-hamburg.de (A.U.); k.rothkamm@uke.de (K.R.); 2 Universität Hamburg and Center for Free-Electron Laser Science (CFEL), Institute for Experimental Physics, Faculty of Mathematics, Informatics and Natural Sciences, University of Hamburg, Luruper Chaussee 149, 22761 Hamburg, Germany; theresa.staufer@desy.de (T.S.); oliver.schmutzler@desy.de (O.S.); ckoernig@mail.desy.de (C.K.)

**Keywords:** X-ray fluorescence, XFI, GEANT4, immune cell, tracking, immunotherapy, gold nanoparticles

## Abstract

**Simple Summary:**

One novel approach in cancer therapy is the use of genetically modified immune cells that are more specifically directed to a tumor than common chemotherapy. This creates the need for medical imaging methods that can be used to track these immune cells during therapy. Our study provides computer simulations of potential applications of X-ray fluorescence imaging for this purpose. We showed that if immune cells were labeled with gold nanoparticles as an imaging marker, the amounts of immune cells that would be expected to be found in a tumor or inflammation site could be detected with our setup. Our feasibility study thus shows results that are promising estimates on what can be achieved.

**Abstract:**

The growing field of cellular therapies in regenerative medicine and oncology calls for more refined diagnostic tools that are able to investigate and monitor the function and success of said therapies. X-ray Fluorescence Imaging (XFI) can be applied for molecular imaging with nanoparticles, such as gold nanoparticles (GNPs), which can be used in immune cell tracking. We present a Monte Carlo simulation study on the sensitivity of detection and associated radiation dose estimations in an idealized setup of XFI in human-sized objects. Our findings demonstrate the practicability of XFI in human-sized objects, as immune cell tracking with a minimum detection limit of 4.4 × 10^5^ cells or 0.86 μg gold in a cubic volume of 1.78 mm^3^ can be achieved. Therefore, our results show that the current technological developments form a good basis for high sensitivity XFI.

## 1. Introduction

A considerably young area of medical imaging is so called molecular imaging, which gives insights into biological and pathological processes that are of great use for research and clinical applications alike. More precisely, molecular imaging is the process of imaging and studying molecular and cellular processes in entire organisms [1]. Not only can it help to deepen our understanding of physiological interactions between cells and their functions, but due to advances in the fields of cell-based therapies and regenerative medicine, the demand for imaging methods capable of visualizing such processes greatly increased in recent years [2,3,4,5]. An important requirement for any imaging method is a high sensitivity, and hence, a reasonable lower detection limit for contrast agents, dyes, or tracers that enables non-toxic imaging of cellular processes in vivo. Current imaging methods capable of detecting sufficiently low concentrations of markers to aid in the research of for example cell-based therapies on the molecular level in vivo include nuclear imaging with Positron Emission Tomography (PET) and Single Photon Emission Computed Tomography (SPECT), Magnetic Resonance Imaging (MRI), and optical imaging [1,6,7]. PET has a good sensitivity for low concentrations of markers, detecting radionuclide concentrations in picomolar ranges, but suffers from intrinsically limited resolution [6,8]. MRI achieves higher spatial resolution at the cost of scanning time and/or sensitivity [9,10]. Optical fluorescence imaging can achieve high spatial resolution and high sensitivity but is greatly limited in depth penetration due to the physical nature of visible light [7,9]. The goal of this work is to further determine the usefulness of a novel imaging method, namely X-ray Fluorescence Imaging (XFI).

XFI is based on the photoelectric effect, the emission of photons with characteristic energy by an atom after its excitation with X-rays. Detecting a signal with this characteristic energy is highly specific for a certain element or marker. XFI for in vivo use on humans was studied in the 1970s already, mainly focusing on the detection of lead in the body [11]. For a variety of elements in the body the in vivo application of XFI in small scanning areas was since studied, including, but not limited to, lead, mercury, cadmium, iodine, or gold [12]. However, clinical in vivo applications of XFI like molecular imaging with clinically available imaging modalities were not developed to date. This is mainly because the biggest problem for scanning big areas in human-sized objects is the high background due to multiple Compton scattering, which prevents fluorescence signals from being detected at reasonable marker concentrations [12,13]. This can, however, be counteracted in several ways, namely the use of a polarized incident X-ray beam, collimation, and by increasing the total detector area [12,13,14,15]. Combining these technical countermeasures with a sophisticated signal analysis like a spatial filtering algorithm to determine the detector area with an ideal signal yield, as previously described [14], helps to further push the limits of XFI to enable in vivo imaging of human-sized objects. Another critical factor for high sensitivity XFI is the choice of X-ray source, as there are different options, all offering individual (dis)advantages and coming with highly varying price tags and sizes. For the analysis of metalloids in biological samples planar scanning with synchrotron-XFI and X-ray Fluorescence Computed Tomography (XRFCT) with synchrotrons or polychromatic X-ray sources are mostly being used currently [16]. Synchrotron-XFI can achieve high spatial resolution in planar images, whereas XRFCT is able to produce 3-dimensional images intrinsically [16,17,18]. The high flux of synchrotron pencil beams enables high resolution scanning at shorter image acquisition times, in contrast to filtered benchtop sources, which make synchrotron-XFI particularly interesting for the application on human-sized objects.

Due to the high cost and very limited access to synchrotrons, numerical feasibility studies for XFI in human-sized objects are needed to optimize the design of practical experiments. For medical purposes, different Monte Carlo-based tools are available, for example PENELOPE, MCNP, and PENFAST are used for dosimetry in radiotherapy, or GATE/GEANT4 and FLUKA are used for medical physics in general [19,20,21]. For the research of radiotherapy with high energy photons, PENH or FLUKA can be used [19,22]. While many toolkits are specific packages designed for different purposes such as planning in radiotherapy or dosimetry in medical imaging, so called general-purpose Monte Carlo codes like GEANT4 offer a wide range of applications [20]. We decided for the commonly used GEANT4 toolkit because it offers high flexibility and supports the modeling of complex geometries [23,24]. Numerous studies evaluated the accuracy of GEANT4 simulations in comparison to practical measurements and other Monte Carlo codes established in medical physics, agreeing that it is suitable for medical research [21,23,25,26]. The practical experience of our team with measurements of reference targets and dosimetry meets the results of these studies, such that the simulation error of Geant4 lies within reasonable limits, e.g., below 6 to 10% [21,23,25]. Furthermore, several GEANT4 simulation studies showed that the sensitivity of XFI in human-sized objects increased in the past decade [13,14,27].

In conclusion, there are several reasons why XFI can be of great use for molecular imaging of human-sized objects: Firstly, in dependance of the tracked material and incident energy, it suffers less from a depth penetration limit than optical fluorescence imaging. Secondly, for any X-ray source, the only limitation to scanning resolution is the scanning beam diameter, enabling microscopic scanning with up to submicrometer resolution in synchrotron-XFI of in-vitro samples [16,28,29]. However, to keep scanning time and radiation dose at a reasonable level, the scanning resolution should always be adapted to the region of interest. For bigger scale in vivo imaging or imaging of human-sized objects, mm^2^ or sub-mm^2^ resolution seems reasonable.

As stated above, for molecular imaging, a high sensitivity, and hence, a reasonable lower detection limit, for any marker has to be achieved. In the case of XFI, choosing the right element to trace is important because it requires both practicability in physical and physiological terms. Elements with a high atomic number such as gold are ideal for XFI because their characteristic X-ray fluorescence energies are high enough to achieve the depth penetration needed for imaging in human-sized objects [27]. Gold proves to be low in toxicity, biocompatible, and chemically stable, and thus is often used for the creation of gold nanoparticles (GNPs) with a wide range of applications such as photosensitizers in radiotherapy or carriers for molecular imaging contrast agents in MRI and fluorescence imaging [30,31,32]. Furthermore, a variety of shapes and sizes of GNPs, affecting their imaging and therapeutic properties, already exist [31,33,34,35]. Therapeutic GNPs for example were loaded into T-cells such that they could be delivered to a tumor site for photothermal therapy in a tumor xenograft-bearing mouse model [36]. Not only could XFI be used to track the delivery of therapeutic nanoparticles in such a case, also the loaded T-cells could be tracked. Cell-based antitumor therapy, for example with CAR-T-cells was shown to be promising for the treatment of acute lymphatic leukemia, yet its application in solid tumors seems to be more challenging [37,38]. Following the injected T-cells is important to not only check if their delivery to the tumor-site was successful, but also to understand the dynamics of the subsequent immune responses [39]. XFI could be used to track such therapeutic T-cells by intracellular labeling with GNPs before their injection. Besides tumor immunology, T-cells play a major role in immune, but also auto-immune responses, such as inflammatory bowel disease (IBD) [40]. In vivo imaging of their distribution and possibly activity at inflammation sites could be helpful to better understand the disease and develop new treatment options. Therefore, as reference to CAR-T-cell therapy and also malignant and inflammatory processes in general, the goal of this work is to simulate GNP-labeled immune cells to examine the application of synchrotron-XFI for gold-labeled immune cell tracking in human-sized objects. Two scenarios, both depending on T-cell tracking, were chosen to act as example for how clinical XFI could be used. These scenarios were simulated in a human voxel-phantom to mimic a real-life application as accurately as possible. To achieve this, it is necessary to estimate how many immune cells, and thus, how much gold would have to be detected to determine the required sensitivity for XFI.

For CAR-T-cell therapy, patients typically receive amounts of 1 × 10^6^ to 1 × 10^7^ cells/kg per injection [41,42]. Using a micro-CT, Meir et al. estimated that 48 h after the systemic intravenous injection of 1.6–2.0 × 10^7^ GNP labeled, targeted T-cells into a tumor-bearing mouse graft, up to 4.6 × 10^5^ T-cells accumulated in the tumor region [43]. An oncological study in patients with metastatic melanoma receiving anti-PD1 therapy showed that the number of CD8+ T-cells in biopsies of melanoma tumor tissue ranged from roughly 500 to 6,500 cells/mm^2^ before treatment and could range from 2,000 to 12,000 cells/mm^2^ after treatment [44]. Assuming that this number of T-cells would accumulate not in a tissue layer of one mm^2^ but a cubic volume of one mm^3^, an amount of 5 × 10^5^ to 1.2 × 10^7^ T-cells per cm^3^, e.g., per roughly one gram of tissue can be extrapolated. Although the latter study provides ex-vivo data of T-cells that were not labeled before, these numbers on their own still indicate the amount of T-cells that are to be expected in a tumor mass, and thus, we estimate the need to be detectable for in vivo tumor T-cell tracking. In the bowel however, a high number of immune cells can be found naturally already, as up to 10^6^ lymphocytes per g of enteric tissue accumulate under physiological circumstances [45]. This number of immune cells may increase drastically during inflammation as for example IBD is characterized by CD4+ T-cell infiltration of the bowel [40]. A minimum number of 1 × 10^5^ − 1 × 10^6^ immune cells per cm^3^ should thus be detectable for immune cell tracking in IBD. The amount of gold per cell is more difficult to determine as it depends on the labeling process as well as on the size and shape of the used GNPs. Also, there is not only a physical limit on how much gold can be taken up by immune cells, but also physiological functions must not be impaired by labeling with GNPs [46].

Usually, cells are labeled with GNPs directly and in vitro using different incubation processes [36,43,47,48]. GNPs could, however, also be delivered to a molecular target in vivo or in vitro by coupling with monoclonal antibodies (mAbs) [49,50,51]. Labeling of T-cells for XFI could therefore either be achieved with in vitro labeling during the preparation of CAR-T-cells or possibly in vivo using antibody-coupled nanoparticles. GNPs complexed with poly-L-lysine and rhodamine B isothiocyanate were used to in vitro label human mesenchymal stem cells with a gold amount of slightly over 600 pg/cell for in vivo tracking with a micro-CT scanner in rats [52]. This work shows in vivo imaging of GNPs that can be achieved using micro-CT in rats already, however, micro-CT is not suitable for human-scale imaging and the measured density of GNPs is not as specific as the signal from X-ray-fluorescence from GNPs. For anti-EGFR antibody-coated GNPs in a tumor model in mice, a labeling efficiency of 3.4 × 10^3^ GNPs per tumor cell, equivalent to 10.52 pg gold/cell, was reached in vitro [53]. For T-cells gold concentrations of up to 195 pg/T-cell were reported [43,46]. Based on this amount of gold per cell, an average concentration of 19.5 μg to 195.0 μg gold per mL, simulating the presence of about 1 × 10^5^ to 1 × 10^6^ T-cells per g tissue, appears realistic and poses a reasonable minimal detectable concentration limit for the setup.

In conclusion, the main goal of this work is to predict the limitations and possibilities of gold-XFI in human-sized objects that can be achieved using a simulated ideal setup, using the example of immune cell tracking. The approaches for background reduction described above are combined in a GEANT4-simulated environment. Thus, a polarized synchrotron pencil beam is used as X-ray source in combination with a 4π-detector and spatial filtering. To examine XFI in human-sized objects, two scenarios were chosen. Firstly, a dilution series of gold in a small group of voxels in the right hemithorax of the voxel phantom was simulated to determine the sensitivity of the setup. For our work, sensitivity is defined as the minimum detectable value of gold concentration or gold amount. The thorax was chosen for this task because CAR-T-cell therapy is an object of research for tumors in the lung [54,55] and because the right lung portion of the voxel phantom is comparably homogeneous, meaning sensitivity would suffer less from location-dependent effects. Secondly, to create a more realistic imaging concept, scanning of several positions in the abdomen of the voxel phantom was simulated with different orientations of the voxel phantom to determine the effect of incident beam angle on sensitivity and radiation dose. The abdomen was chosen because of its higher level of inhomogeneity. In reference to IBD, in this scenario gold was added to the colon tissue, as XFI could help to better understand such auto-immune diseases by immune cell tracking.

## 2. Materials and Methods

### 2.1. Software and Setup

GEANT4 is a software toolkit for the simulation of particles passing through matter and is well established in particle, nuclear, and medical physics [56]. These Monte Carlo simulations are an important aid in the research of XFI due to the limited access to synchrotron facilities, and especially because experiments on humans like those simulated here are not practical yet. The simulations were run using the version 4.10.05.p01 and the physics model polarized Livermore.

The International Commission on Radiological Protection (ICRP) created a male and a female adult reference computational phantom, further referred to as voxel phantom, based on CT scans of real patients to allow for radiation dose calculation and the examination of radiation protection based on realistic anatomy [57]. Because of its higher resolution, the female voxel phantom was used. The voxel dimensions were 1.775 × 1.775 × 4.85 mm, in a volume of 137 × 299 × 346 voxels, in accordance with ICRP-publication 110 [57]. The voxel phantom was built in GEANT4 using the details for the elemental composition of any tissue provided with the voxel phantom by the ICRP. Labeling of tissues was achieved by adding gold to those predefined elemental compositions.

**Figure 1:**
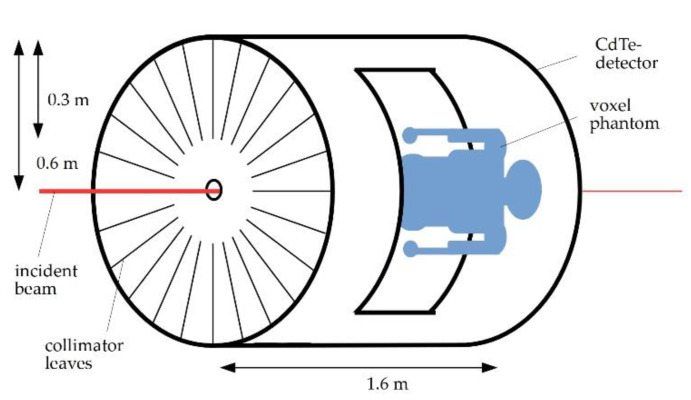
Schematic of simulated setup. 4π-detector consists of Cadmium-Telluride and its Collimator consists of 3600 Molybdenum leaves. Detector has form of a cylinder, with holes on both sides to fit voxel phantom in. In this case, holes are 26.75 × 106.45 cm wide to enable sidewards mobility of phantom for scanning several positions. Red line shows incident pencil-beam with a diameter of 1 mm, radially extending lines show a simplified arrangement of collimator leaves. Blue shape illustrates how voxel phantom is placed inside tube such that beam axis extends sagittally through it.

The detector was built using the GEANT4 sensitive-detector geometry with a 1.0 mm thick layer of cadmium telluride as detector material and a detector resolution of σ = 300 eV (rms). Both the true and the recorded energy was saved for further data analysis. The recorded energy takes detector effects such as hole tailing and efficiency into account and provides more realistic results, whereas the true energy provides the exact energy of every photon that hit the detector. For all simulations, a zylindrical 4π-detector with a radius of 0.6 m and a length of 1.6 m was created. Caps were added to the top and bottom of the cylinder to increase the detector surface. At both sides, holes for the voxelphantom were cut in, with their size depending on the orientation of the voxel phantom. For scans where the voxel phantom lied on its side, they were 26.75 × 106.45 cm wide; for scans where the voxel phantom lied on its back, they were 58.38 × 48.64 cm wide. This ratio of twice the dimension of the voxel phantom in the direction of movement for the scan and 1.1-times the dimension orthogonally to it were chosen to allow for full mobility during a scan of the voxel phantom without letting its volume collide with the detector. Also, holes of the size of the incident X-ray beam were cut into the caps to let it enter and leave the detector without interaction. As for the incident X-ray beam, a monoenergetic polarized pencil-beam with a diameter of 1 mm^2^ was used. Figure 1 represents a schematic of the simulated setup. Based on the work by Grüner et al., an energy of 85 keV was chosen for the incident X-ray beam as this energy lies above the K-edge of gold and does work well with their spatial filtering algorithm [14]. Choosing this energy results in several K-shell fluorescence lines such as K_α1_ at 68.80 keV and K_β1_ at 77.98 keV, among others [58]. To keep the radiation dose at a reasonable level, an amount of 10^9^ incident photons was simulated for each experiment. One method to achieve background reduction for better signal yield is using a radial collimator, which ranges from an inner radius of 0.3 m to the detector’s outer radius of 0.6 m and consists of 3600 molybdenum leaves. The second method is spatial filtering of the fluorescence signals to determine the detector areas with optimal signal-to-background ratios.

### 2.2. Procedure

**Figure 2:**
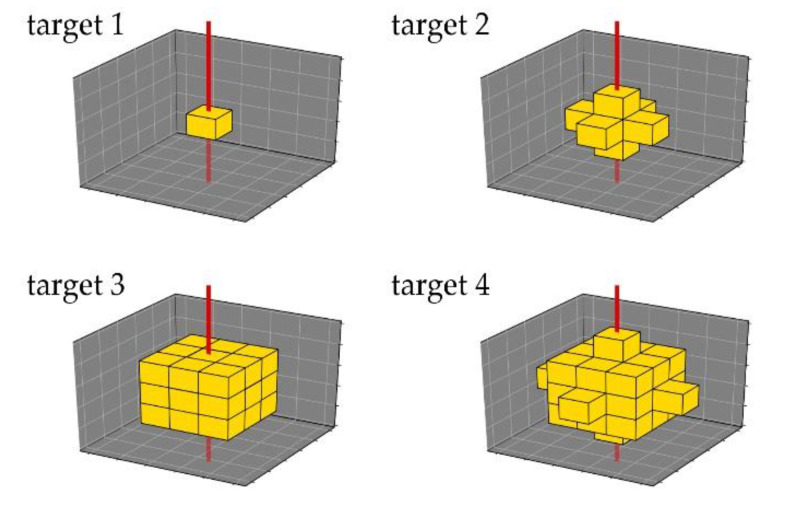
All four different targets to simulate XFI-scans in the thorax. Shapes shall represent a tumor or inflammation at different sizes and consist of voxels of lung-tissue, to which certain amounts of gold were added. In reality, such an area would have a round shape; targets follow voxel geometry of voxel phantom, and are thus cubic, depending on their size. Incident beam (**red**) hits them orthogonally, thus between target 2 and target 3, amount of target voxels in beam volume does not change.

XFI-simulations in the thorax were created for 4 different targets, consisting of the lung tissue defined by the ICRP and containing gold at different concentrations, which will be further referred to as lung targets. Those lung targets were placed centered in the right lung. The lowest gold concentration per voxel was set to 1.0 μg/mL and the highest was set to 10.0 mg/mL. 1.0 μg/mL was chosen as the lowest concentration because Grüner et al. showed how for a sphere of water with similar dimensions as the voxel phantom, it could be detectable using a similar setup like this one [14]. In the simulations, the concentration was increased stepwise from 1.0 μg/mL to 0.01 mg/mL, 0.1 mg/mL, 1.0 mg/mL and 10.0 mg/mL. The shapes of the targets were chosen to model different sizes of a tumor or inflammation site where labeled T-cells would accumulate. Figure 2 shows the increase in size by one layer of voxels for each target. The incident beam hit the voxel phantom orthogonally from the front only.

**Figure 3:**
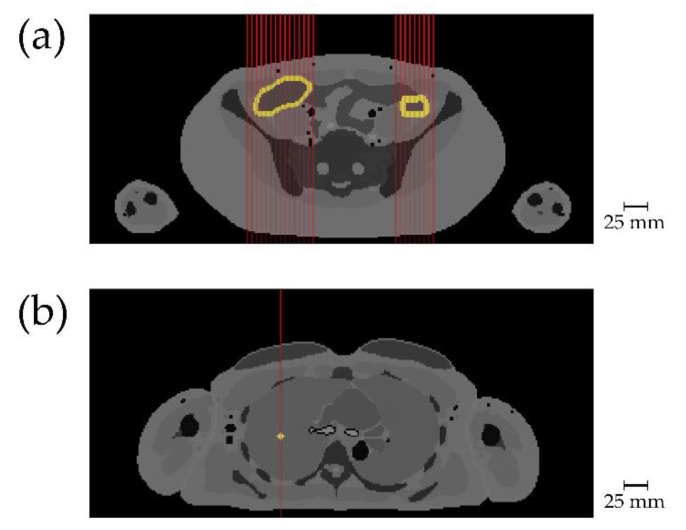
Scanning positions with target locations. Target tissue with added gold is shown in light yellow, red lines indicate incident X-ray beams. (**a**) Slice 203 of voxel phantom, entire colon, hence both right and left colon contained gold for scan. Only incident beams for scan from front are shown; (**b**) slice 277 of voxel phantom with target 4 in right lung. Incident beam shows that here only a single point is scanned. Scale bar in mm, note that one voxel is 1.78 × 1.78 mm wide in this view.

Scanning of a slice in the abdomen was simulated in several steps. In a prescan of just one position (70 mm right of the center of the voxelphantom) like in the thorax, descending concentrations of gold were simulated similar to the simulations in the thorax, with the incident beam hitting the voxel phantom from the front. The voxels consisting of the media “Large-Intestine”, hence, the colon, were filled with the same amounts of gold as the targets in the right lung. A total of 1.0 mg/mL gold was then chosen as gold concentration for the actual scan as it delivered a clearly detectable signal. Scanning of the abdominal slice was simulated with three incident beam angles, hitting the voxel phantom orthogonally from the front, back, and right-side. See Figure 3 for the location of the gold targets and the incident beam angles. The scan from the front started slightly next to the right colon at 100.0 mm right of the center of the voxel phantom, proceeding to 30.0 mm right of the center in 5.0 mm steps and started again slightly next to the left colon at 60.0 mm left of the center of the phantom, proceeding to 100.0 mm left of the center in 5.0 mm steps. Hence, a total of 24 scanning positions resulted at the location of right and left colon. The scan from the back was simulated at the exact same positions. The scan from the right side started 5.0 mm above the center of the voxelphantom, proceeding to 60.0 mm above the center of the voxelphantom in 5.0 mm steps as well. Because from this perspective the right and left colon overlap, a total of only 12 scanning positions resulted. Because the voxelphantom already provides the exact location of the colon, calculation time was saved by not simulating the entire abdominal slice. This, however, well suits our intent to simulate an ideal setting, as to reduce scanning time and dose, comodal imaging with, for example, sonography, could help finding the ideal scanning position or region of interest in advance. This would rule out the need to scan the entire body section.

### 2.3. Statistical Analysis

In practice, the fluorescence signal needs to be discriminated from the background in an energy spectrum measured by the X-ray detector. This is done by fitting functions to the fluorescence peaks and the background separately, such that the amount of background photons can be subtracted from the total measured photons, yielding the estimated amount of signal photons. To rule out possible errors from this process, for our study, the significance (Z) of the fluorescence signal over the background was calculated directly from the simulated spectra. This was done for the K_α_ region, as well as the K_β_ region, before and after applying the spatial filtering algorithm. This way, a level of significance for any signal can be defined, above which it can be considered detectable, independent of the need for fitting functions. As described by Grüner et al., this significance depends on the statistical *p*-value, the probability of a detected signal being taken as such, despite only being a background fluctuation [14]. It is calculated using the amount of fluorescence signal photons and background photons in a range of ±3σ of the detector resolution around the fluorescence peaks. In accordance with previous studies and practical experience of our team, a significance value of 5σ for a fluorescence signal was defined as the minimum significance value to discriminate signal over background even without detailed analyses during measurements. Here, a high significance means a high sensitivity of a setup, as it derives from a fluorescence signal that is high enough to be detected above the background. For significance calculation the recorded energy is used, as this detector output is closer to reality than the true energy.

To determine the subset of such detector pixels whose summed spectra show the highest significance yield, the spatial filtering algorithm from Grüner et al. was applied [14]. In contrast to their work, where the detector area was divided into many pixels, in this work the detector area was divided only into 16 × 16 detector panels, each with unique counts of signal and background photons. To determine the combination of detector panels which yields the highest significance, the significance of the fluorescence signal was calculated 256 times, each time using all panels but one, so that every panel was left out once. Then, the panel, of which the exclusion led to the highest significance for all others combined, was deleted. This means the removal of the panel with the worst signal to noise ratio, that thus reduces the combined significance the most. This process of deleting panels and calculating the significance for the left ones was repeated until the significance of the remaining panels could not be improved further.

A linear regression of the calculated signal significance for both K_α_ and K_β_ fluorescence as dependent and the gold concentration as independent variable was done for any target in the right lung as well as the gold-labeled colon in the abdominal prescan. Solving the trend function for y = 5, hence calculating the gold concentration that results in a signal significance of 5 and therefore the minimum detectable value, provides our estimations for the lowest detectable gold concentration for any given setup.

Radiation doses were calculated directly from the energy deposition in all voxels of each tissue, yielding the tissue- and organ doses, and the energy deposition in all voxels at the beam position, delivering the dose in the beam volume. For every setting, the average dose was calculated by dividing the sum of all doses by the number of all doses, providing the arithmetic mean (*M*). The corresponding standard deviation (*SD*) was calculated as well, as it is an indicator for how dispersed the doses are in relation to their arithmetic mean, which is especially important for tissue doses when scanning different positions like in the abdominal scan.

## 3. Results

### 3.1. Thorax

#### 3.1.1. Detectable Gold Concentrations, Extrapolation of Sensitivity

**Figure 4:**
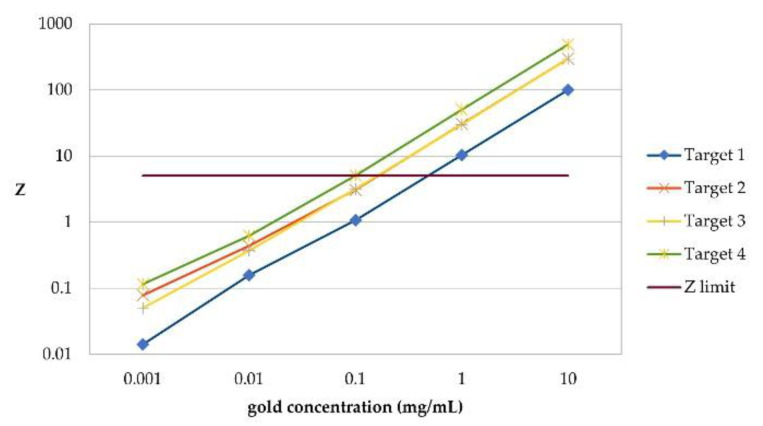
Signal significance (**Z**) per gold concentration for every lung target when scanning thorax. Each target is a group of gold-filled voxels in right lung. Its size increases with its number. For target 2 and 3, scanned volume contains 3 gold-filled voxels, and for target 4 it contains 5, respectively. Significance for Target 2 and 3 differs at lowest gold concentration despite same amount of gold-filled voxels in scanned volume, most likely due to a reduced efficiency of spatial filtering at an overall low significance. Z limit of 5 was defined based on preliminary studies of our research group and is lowest significance at which a signal can be detected above background Here, significance calculated for K_β_ fluorescence after application of spatial filtering algorithm is shown because for K_β_, a higher significance than for K_α_ could be found.

See Figure 4 for the K_β_ signal significance that can be calculated after spatial filtering for every lung target and at every gold concentration. An amount of 10.0 mg/mL gold per voxel was detectable at any target size with and without using the spatial filtering algorithm. For the amount of 1 mg/mL gold per voxel, any target could be detected when applying the spatial filtering algorithm. Without it, target 1 is not detectable when analyzing for K_α_ and targets 1 to 3 are not detectable when analyzing for K_β_. None of the targets is detectable at a lower gold concentration. Using a linear regression analysis for the results for any gold concentration per target, the best sensitivity is estimated for target 4, with a minimum detectable concentration of 92.24 μg/mL gold per voxel when filtering for K_β_ and 96.35 μg/mL gold per voxel for K_α_, respectively. The lowest sensitivity is estimated for target 1, e.g., a single voxel, where an amount of 487.05 μg/mL gold would be detectable when filtering for K_β_, and 503.3 μg/mL gold when filtering for K_α_. The sensitivity for target 2 and 3 lies in-between, with a minimum of 164.12 μg/mL (K_α_) and 161.21 μg/mL (K_β_) for target 2 and 163.96 μg/mL (K_α_) and 164.02 μg/mL (K_β_) for target 3 are estimated to be detectable.

#### 3.1.2. Radiation Doses

**Figure 5:**
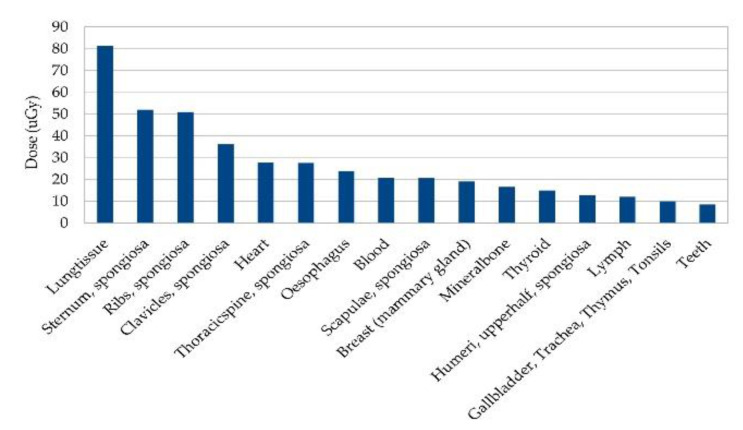
Estimated organ dose deposition for the 16 tissues with highest average dose for scanning of an area of one cm^2^ with one mm^2^ resolution at thorax. This is estimated for a total of 100 scanning positions, as doses are averaged over all scans with all gold concentrations and target sizes for one simulated scan point, and then multiplied by 100.

The beam doses for all targets range from 2.18 mGy (1 μg/mL gold, target 1) to 2.33 mGy (10.0 mg/mL gold, target 4) per shot, with the higher doses being measured at higher concentrations of gold and bigger targets, respectively. The average beam dose per shot is M = 2.20 mGy, standard deviation SD = 0.04 mGy. The full body doses range from 67.59 nGy (0.1 mg/mL gold, target 1) to 69.84 nGy (10.0 mg/mL gold, target 4) per shot, with an average body dose of M = 68.03 nGy, SD = 0.58 nGy per shot. The average tissue doses are highest for the lung tissue, followed by the tissue ‘sternum spongiosa’. The lowest doses per shot are detected in the tissues ‘lower leg bones spongiosa’ and ‘lower leg bones medullary cavity’. For the thorax scans, however, just a single shot would not be enough to localize a tumor with an unknown location. Even if its position would be known a priori, several points would be needed to examine the size and contours of the tumor. See Figure 5 for an estimation of the 16 tissues with the highest dose uptake when scanning an area of 1 cm^2^, with a total of 100 points for a 1 mm^2^ resolution.

**Figure 6:**
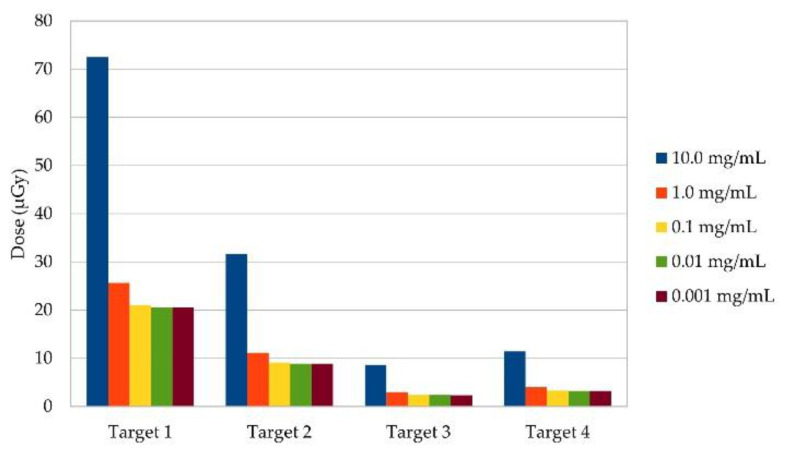
Dose deposition per shot in lung targets depending on gold concentration and target size. Targets are groups of gold-filled voxels in right lung, and their size increases with their number. Dose is higher for higher gold concentrations, due to more absorption because of density of lung targets. With bigger size of target, dose is lower because it also depends on the mass of lung targets. However, for target 4, doses are higher than for target 3, as increase in voxels in beam volume outweighs increase in total mass of target.

As can be seen in Figure 6, for any given gold concentration, the dose per target is lower with increasing target size for targets 1, 2, and 3, with a slightly higher dose for target 4 over target 3. This shows the direct dependence of dose on mass in the beam volume and mass of the target in general. For all targets, most of the increase in size happens outside of the beam volume. Therefore, despite more mass in the beam volume for target 2 and 4, only for the latter a slightly higher dose than in target 3 results, whereas for all other targets, the dose reduces with increasing target size. The gold concentration in the target appears to have the effect of increasing dose absorption through higher amounts of gold, with its effect not depending on target size. 

### 3.2. Abdomen

#### 3.2.1. Detectable Gold Concentrations, Extrapolation of Sensitivity

**Figure 7:**
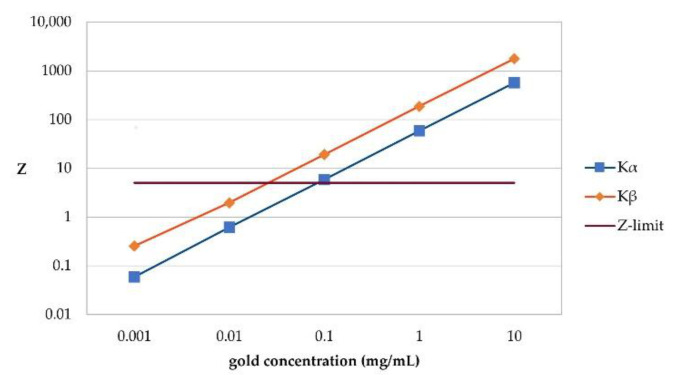
Signal significance (**Z**) per gold concentration in abdominal prescan. Significance for both K_α_ and K_β_ after application of spatial filtering algorithm is shown. Z-limit of 5 like in scan of thorax indicates at which significance a signal could still be detected and is set based on preliminary studies of our research group. Therefore, its crossing point with K_α_ and K_β_ marks lowest detectable gold concentration for each, respectively. Like in thorax, filtering for K_β_ results in higher significance than for K_α_. This results from higher efficacy of our spatial filtering algorithm for K_β_-signal region, as background there mostly derives from single Compton scattered photons.

After scanning the colon from the front, the tissue large intestine was filled with the same amounts of gold as the lung target, and 10.0 mg/mL gold, as well as 1.0 mg/mL gold, were detected with and without using the spatial filtering algorithm if analyzing for K_α_. When analyzing for K_β_, 10.0 mg/mL, 1.0 mg/mL and 0.1 mg/mL gold with filtering, but only 10.0 mg/mL and 1.0 mg/mL gold without the spatial filtering algorithm are detectable. See Figure 7 for the K_β_ signal significance at every gold concentration in this abdominal prescan. The linear regression analysis of these results suggests that when filtering for K_α_, a minimum of 79.63 μg/mL gold and when filtering for K_β_ a minimum of 14.15 μg/mL gold can be detected using our setup.

#### 3.2.2. Dependence on Incident Beam Angle

**Figure 8:**
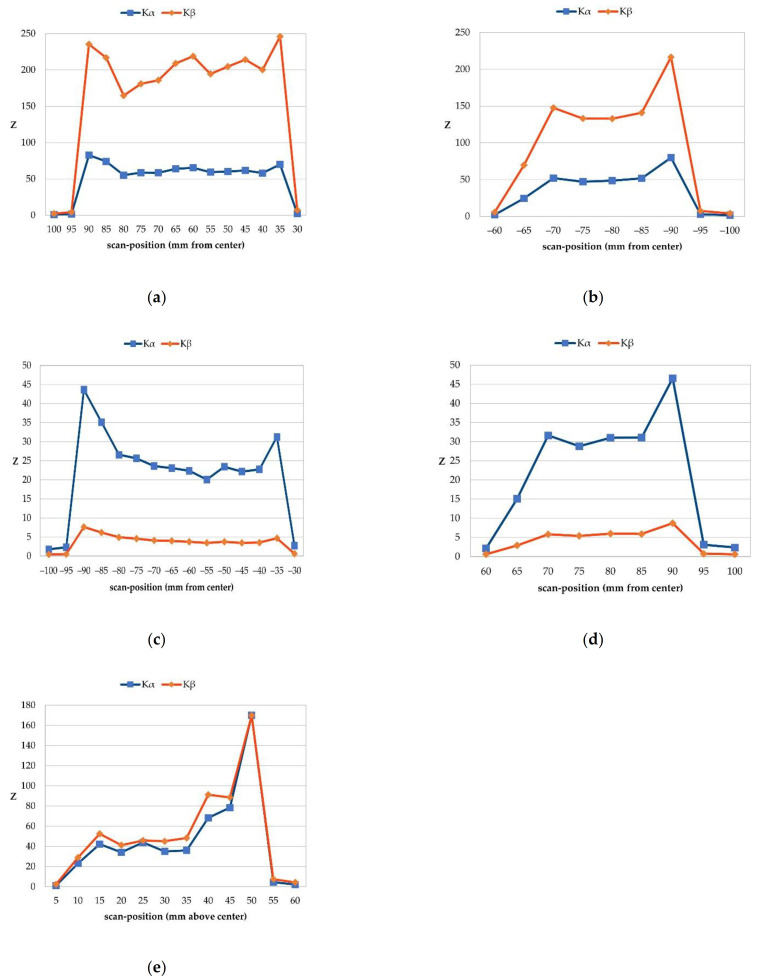
Signal significance (Z) after spatial filtering for both K_α_ and K_β_ fluorescence at all scanning positions in abdominal slice: (**a**) significance per position at right colon when scanning from front; (**b**) significance per position at left colon when scanning from front; (**c**) significance per position at right colon when scanning from back; (**d**) significance per position at left colon when scanning from back; (**e**) significance per position at both right and left colon (overlapping) when scanning from side. Spikes at edges of any graph indicate higher amount of gold in beam volume, hence they show that here, colon walls are hit straightly. Subsequently, uneven curves do not arise from uncertainties in significance (which is smaller than marker size), but rather from varying scanning conditions at different scanning points, such as different tissues lying in scanning volume.

Scanning the voxel phantom with three incident beam angles shows an impact on signal significance and radiation dose. When filling the colon with 1.0 mg/mL gold, the signal is detectable in any scan where the beam volume contained gold-filled voxels for both K_α_ and K_β_, with and without the spatial filtering algorithm, when scanning from the front and from the right side. Scanning from the back shows that this is only the case for K_α_ when applying the spatial filtering algorithm. Analyzing for K_α_ without it only shows a detectable signal at very few positions such as 85 mm right to the center of the voxel phantom or 90 mm to the left, where more gold-filled voxels are located in the beam volume. Analyzing for K_β_ shows no detectable signals at any position without using the spatial filtering algorithm. Using it shows only few at 90 and 85 mm right to the center of the voxelphantom, and 90 mm to the left, where again more gold-filled voxels are located in the beam volume than at the other positions. As can be seen in Figure 8, in general, a higher significance is measured at the edges of the scanning positions, as there are more voxels containing gold and less bones are located in the beam volume (Figure 8a, for example Z being 235 [position 90 mm] and 246 [position 35 mm] for K_β_ fluorescence). For the scan from the right side, the highest significance is detected at 50 mm above the center of the phantom with Z being 170 for both K_α_ and K_β_ fluorescence. In conclusion, from the front, the K_β_ significance is higher than for K_α_, which shows a higher efficiency of spatial filtering for K_β_ in this scenario. However, from the back the K_α_ significance is higher, most likely due to the overall lower significance per position, which reduces the efficiency of the filtering algorithm. The significance for both K_α_ and K_β_ are comparably high from the side. This indicates that the distance in between the incident beam hitting the phantom and hitting gold-filled voxels, which is highest from the back and lowest from the front, does not only impact signal significance, but also the efficiency of spatial filtering.

#### 3.2.3. Radiation Dose

The incident beam in our setup does not only have an impact on signal yield, but also on radiation dose. Naturally, both factors depend on the scanning position, as this results in different tissues being located in the beam volume, and the incident angle because this determines the order and extent at which the tissues are hit, attenuating the beam differently depending on the tissue’s density. The beam dose in the prescan of the abdomen from the front ranges from 1.85 mGy (1.0 μg/mL gold in the tissue ‘Large intestine’) to 1.90 mGy (10 mg/mL gold in the tissue ‘Large intestine’). The average beam dose is *M* = 1.86 mGy, *SD* = 0.02 mGy. The full body dose ranges from 91.45 nGy (1.0 μg/mL gold in the tissue ‘Large intestine’) to 93.53 nGy (10 mg/mL gold in the tissue ‘Large intestine’) with an average full body dose of *M* = 91.91 nGy, *SD* = 0.91 nGy. In the entire abdominal scan from the front, the full body dose ranges from 75.41 nGy (95 mm right to the center of the voxelphantom) to 95.24 nGy per position (55 mm right to the center of the voxelphantom). The average full body dose per position is *M* = 87.15 nGy, *SD* = 6.42 nGy and the total full body dose is 2.09 μGy. When scanning from the back the full body dose ranges from 64.96 nGy (100 mm right to the center of the voxelphantom) to 91.39 nGy per position (45 mm right to the center of the voxelphantom), with an average full body dose of *M* = 79.06 nGy, *SD* = 9.11 nGy and a total full body dose of 1.90 μGy. When scanning from the right side the full body dose ranges from 64.85 nGy (60 mm above the center of the voxelphantom) to 89.44 nGy per position (35 mm above the center of the voxelphantom), with an average full body dose of M = 80.27 nGy, SD = 8.33 nGy and a total full body dose of 963.27 nGy. The average beam doses per shot for simulations in the thorax and abdomen are comparable, whereas the beam dose in the thorax with 2.20 mGy lies a bit higher than the abdominal one with 1.86 mGy. This can be explained with the lower density, and thus, weight of lung tissue, which makes up most of the voxels scanned in the thorax. The average full body doses per shot of 68.03 nGy in the thorax and 87.15 nGy in the abdomen also lie in a comparable range, whereas here the abdominal dose is a bit higher. This most likely results from the higher amount of more dense voxels in proximity to the beam, as they take up more dose from scattered radiation.

**Figure 9:**
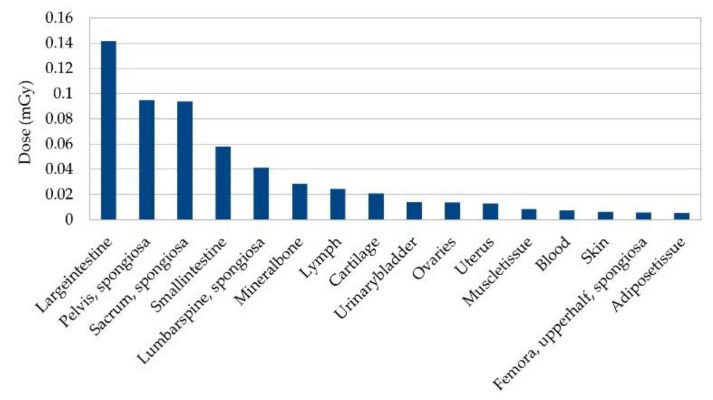
Organ dose deposition for 16 tissues with highest dose for scan of a slice in abdomen. This is estimated for a total of 120 scanning positions (24 simulated positions times 5), only located at a certain region of interest. It is thus to be understood as best case scenario, where almost no irrelevant positions were scanned.

Another important aspect is the tissue doses summed up for all scanning positions. For a total of 24 simulated scanning positions when scanning from the front, the highest dose uptake is observed in the tissue ‘Large intestine’ filled with 1.0 mg/mL gold, with a total dose of 28.31 μGy, which is followed by the tissue ‘Pelvis spongiosa’ with a total dose of 18.96 μGy. The lowest doses are detected in the tissue ‘Lower leg Bones medullary cavity’ with a total dose of 0.12 nGy and the brain with 0.20 nGy. Figure 9 shows an estimation for the 16 tissues with the highest estimated doses if the 24 scanning positions are multiplied by 5, hence if the colon would be scanned with 1 mm scanning steps. The amount of 24 scanning positions for scanning from the front is a simplified setup, similar to the one-position-only scans in the thorax. Even when assuming a 1 mm^2^ resolution, the estimated doses still represent an idealized setup where the scanning positions of interest were known a priori. In reality, more scanning positions per slice, and thus, a higher summed dose, are to be expected.

When scanning from the back, for a total of again 24 scanning positions, the highest dose is detected in the tissue ‘Sacrum spongiosa’ with a total dose of 27.62 μGy. The second highest dose is observed in the tissue ‘Pelvis spongiosa’ with a total dose of 22.99 μGy. The lowest dose is measured in the tissue ‘Lower leg bones, medullary cavity’ with a total dose of 0.128 nGy, as well as the brain with a total dose of 0.20 nGy. When scanning from the right side, for a total of 12 scanning positions, the highest dose is detected in the tissue ‘Large intestine’ with a total dose of 10.61 μGy. The second highest dose is observed in the tissue ‘Pelvis spongiosa’ with a total dose of 9.55 μGy. The lowest dose is measured in the tissue ‘Lower leg bones, medullary cavity’ with a total of 63.00 pGy, as well as the brain with a total dose of 138.03 pGy. The different tissues with a highest dose uptake directly result from the incident beam angle, as this defines which tissue with a high density will be hit first and thus will take up most of the dose.

## 4. Discussion

The main goal of our work was to determine whether gold-nanoparticle based XFI could be used for clinical immune cell tracking in human-sized objects. This was studied with two scenarios, one in the thorax and one in the abdomen, with regards to sensitivity, radiation dose, and resolution. 

### 4.1. Sensitivity in Thorax and Abdomen

The highest sensitivity when scanning the thorax was found for the biggest target (target 4) with 92.24 μg/mL gold for 5 voxels in the beam volume, whereas the lowest sensitivity was found for the smallest target (target 1) with 503.3 μg/mL gold for one voxel in the beam volume. In the abdominal scan where 6 to 8 voxels containing gold are located in the beam volume, the minimum detectable gold concentration was found to be 14.15 μg/mL. One voxel has a volume of 15.28 mm^3^. Multiplying this volume with the lowest concentration of gold for target 1 in the lung results in a detectable amount of 7.31 μg gold in said volume. Considering the beam area of 1 mm^2^, the beam volume actually containing gold, 1.78 mm^3^ was even lower, resulting in sensitivity for a gold amount of 0.86 μg in 1.78 μL. This lies well in the range of the estimation by Grüner et al., where 1.2 μg gold in a 1 mm diameter spherical target, thus a volume of 0.52 mm^3^ or <1 μL, was detectable in a 30 cm diameter water sphere [14]. Based on T-cell labeling efficiency, we estimated that a sensitivity for 19.5 μg to 195.0 μg per mL, hence 1 × 10^5^ to 1 × 10^6^ T-cells per ml would be required for T-cell tracking. If the incident beam hit such a concentration of T-cells in a volume of 1 mL, thus scanning 1% of that volume when using a 1 mm^2^ wide beam, an amount of 0.20 μg to 1.95 μg gold would be located in the beam volume. Using our geometry, this would be possible for an amount of roughly 4.4 × 10^5^ T-cells, assuming a setup like target 1 in the thoracal scan with a sensitivity for 0.86 μg in the beam volume. Immune cell tracking could thus be achieved assuming a homogenous distribution of T-cells in a volume bigger than a single voxel. However, the natural distribution of T-cells in any setting may vary. Also, if for CAR-T-cell therapy only an amount around 1 × 10^6^ to 1 × 10^7^ is injected, it is likely for them to spread in their target region, thus reducing their number per ml below that amount. In the work by Tumeh et al. [44], an amount of up to 1.2 × 10^7^ T-cells per cm^3^ tumor tissue was observed. As these T-cells were not injected but a part of the physiological antitumor response, labeling with GNPs would have to be achieved in vivo, possibly using monoclonal antibodies. If this way a similar labeling efficiency as with in vitro labeling could be achieved, the T-cell amount would lie within the detection limit of our setup. 

**Figure 10:**
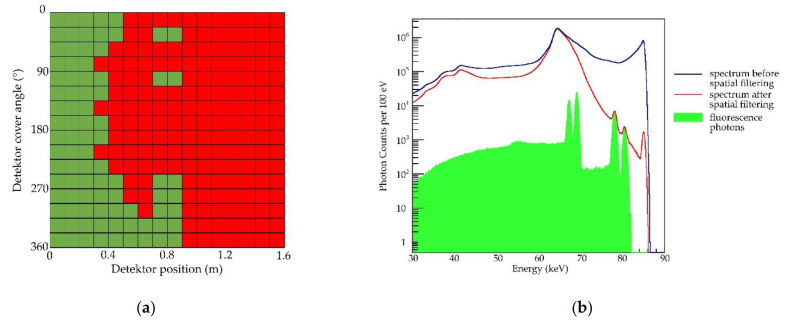
Effects of spatial filtering for K_β_ fluorescence in abdominal prescan for 1.0 mg/mL gold in large intestine: (**a**) Detector panels selected (**green**) and deleted (**red**) for significance calculation after spatial filtering. Cylindrical detector area is spread flat, thus angular position of a tile is plotted against its position over long side of the detector. For K_β_, mostly backward panels are used for significance calculation because of a smaller background from single Compton scattered photons there; (**b**) corresponding energy spectra before and after filtering. In green, contribution of all fluorescence photons to total spectrum is shown. After spatial filtering, K_β_ peaks are visible, whereas the background in K_α_ region is not decreased enough to make fluorescence photons detectable.

The sensitivity of our setup resembles the estimates after applying the spatial filtering algorithm similar to Grüner et al. [14]. It is a bit lower, which most likely mainly arises from two major differences in the simulated setups. Firstly, creating a wide hole in the X-ray detector for the voxel phantom leads to a loss of detector parts with very high sensitivity [12,15]. Secondly, the voxel phantom is a more complex geometry than a sphere consisting of water. As the remaining, most sensitive X-ray detector parts are located at the sides of the voxel phantom, the fluorescence signals have to partly cross the bones of the upper arms at both sides before entering the detector. Due to the higher density of the bones, this results in more signal attenuation than when the signal just spreads in a water sphere [59]. This effect can also be observed in the abdominal scan. As scanning with three different incident beam angles showed, the lowest significance per position is detected when scanning the gold-filled colon from the back. Not only do the signal photons arise in a region of the voxel phantom that is mostly surrounded by bones, namely the pelvis and sacrum, but also the incident beam gets attenuated when crossing the sacrum bone before it can hit the gold-filled colon, when scanning from the back and from the side. As the energy of the incident beam lies closely above the K-edge of gold, only slight attenuation is enough to lower the energy of an incident photon below the K-edge. This can be well seen in the significance per position when scanning from the side, where on the position without bones in the beam volume (50 mm above the phantom center) the highest significance is calculated. Furthermore, the longer distance between incident beam and colon when scanning from the back has an effect, similar to the depth limitation of excitation light sources in optical imaging, which results in attenuation of the beam in general. Our results show another effect of this distance, however. It is therefore necessary to take a look at the efficiency of the spatial filtering algorithm. Figure 10 shows the deleted detector panels with the corresponding energy spectra before and after filtering for K_β_ at one position in the abdominal scan.

As the abdominal scans showed, for scanning from the front, spatial filtering for K_β_ yielded a higher significance than for K_α._ Note that the decrease in background photons after spatial filtering in the work by Grüner et al. was located in the K_α_ region, as there the K_α1_ and K_α2_ peaks were used for spatial filtering [14]. The difference between K_α_ and K_β_, the higher significance after spatial filtering of signals in the scan of the thorax and abdomen from the front can be explained by the nature of the spatial filtering algorithm. It makes use of the spatial anisotropy of the single Compton scattered photons when using a polarized incident beam, which make up most of the background photons with an energy close to the incident beam energy. The K_β_ fluorescence has a higher energy than K_α_ fluorescence and lies closer to the incident beam energy, and therefore the spatial filtering algorithm has a higher effect on improving signal significance for K_β_. In general, the single Compton scattered photons have a higher energy at the forward detector panels, and thus increase the background there more at the K_β_ region than at the K_α_ region. In the backward detector panels, their energy is lower and thus they increase the background there more at the K_α_ region than at the K_β_ region. However, the abdominal scan from the back where filtering for K_α_ yielded a higher significance than for K_β_, shows that not one of both exclusively is better. One likely explanation is that the Compton background on the backward panels in this scenario is higher in relation to the fluorescence signals, due to the longer distance the incident beam travels in the voxel phantom before hitting the gold-filled voxels of the colon. Therefore, the forward panels yield a higher significance; hence, K_α_ significance is higher than K_β_ significance. This would also explain why the work by Grüner et al. showed a higher efficiency of spatial filtering for K_α_, because the gold target there was located in the center of a 30 cm diameter sphere [14], hence a setup more comparable to scanning the abdomen from the back than from the front. For this work, with 16 x16 detector panels we divided our detector into fewer panels than in their work, which avoids artifacts among the selected detector panels, but might lead to an underestimation of the highest possible significance due to the lower resolution of the panel layout. Nonetheless, our comparable results with regards to sensitivity show that this measure is reasonable.

In conclusion, our study suggests that for any anatomic region and target tissue, a different setup of incident beam angle and/or scanning direction might be optimal. For possible clinical XFI this means that there would be no ‘one-fits-all’ approach with our setup. A major challenge here is that currently only synchrotrons are able to produce brilliant X-ray beams with a reasonable flux, where changing the incident beam angle to any direction is fairly difficult. Moving and tilting a patient into every direction and angle is not possible, however. Therefore, more compact X-ray sources like laser-based Thomson scattering sources could be an ideal solution [60,61,62,63].

### 4.2. Effects of Target Size

With synchrotron sources high spatial resolution can be achieved, as it is only defined by the applied beam diameter. Our results when scanning the lung target show this very well. The minor difference in significance between targets 2 and 3 shows how for a pencil beam with an energy close to the K-edge of gold, the major part of fluorescence results from gold in the beam volume, whereas the added gold-filled voxels of target 3 hardly contribute to the signal. However, this also means that the sensitivity for detecting a tumor mass or inflamed area does depend on how it is hit or targeted. If it were to be wide in its expansion yet flat in relation to the beam axis, it would be harder to detect. Also, a more diffuse process would not be as easy to detect as a localized one that concentrates more gold in a smaller volume, which can be seen in the significance spikes of the fluorescence signals at the edges of the abdominal scans. This also results from the direct dependence of signal strength on gold in the beam volume as at these positions the colon is hit straight through its entire wall rather than twice in a small portion, which results in more voxels containing gold in the beam volume.

### 4.3. Dose in Thorax and Abdomen

Not only is the signal induced, but also radiation dose depends on the incident beam angle. The scanning position results in different organs and tissues located in the beam volume, thus determining direct and indirect exposition. Generally, in medical imaging, a principle is to keep the area exposed to radiation to a minimum and to keep organs at risk out of the direct exposure zone. Therefore, our colon scan, where just the regions of interest were scanned, could be close to clinical reality. It is important to consider the dose for any tissue when doing an entire scan of one or several slices. As for the doses per slice given above, these are calculated for 5 mm-steps and when only scanning the regions of interest within a slice. To estimate the total dose for a 1 mm^2^ resolution, e.g., 1 mm steps, either the dose for any position is multiplied by 5 and added up or the total dose is directly multiplied by 5. This is closer to reality than just multiplying the average dose per position by the number of scanning positions for any given resolution. This way, the tissue doses would naturally be roughly 5-times higher, e.g., result in a dose of 0.14 mGy for the tissue large intestine and 0.10 mGy for the pelvis spongiose for example, which had the highest dose uptake. Scanning a total of 10 slices would result in a 10-fold increase of dose, thus resulting in 1.42 mGy for the tissue large intestine and 0.95 mGy for the pelvis spongiosa.

### 4.4. Comparison with Other Imaging Modalities

Our setup could be used to create 2D images through planar scanning. Parallel anatomic imaging for location of the detected signals could provide additional information, as attenuation correction could help improve quantitative signal analysis in a similar manner to optical imaging and PET [64,65]. Comodal imaging with for example CT or MRI would be one possibility. 

When comparing our XFI setup for human-sized objects to currently available clinical molecular imaging methods, several up- and downsides exist. Firstly, resolution in the mm^2^-range can be achieved and is only limited by the incident beam size. For clinical scale this compares well with the high resolution of ca. 1 mm^2^ in MRI and conventional CT, whereas optical imaging (1–5 mm^2^) and nuclear imaging (5–10 mm^2^) lie behind [6,7]. Secondly, sensitivity for micromolar to nanomolar gold concentrations can be achieved in our simulations, as the lowest detectable amount of 0.86 μg gold in the thorax scan equals 4.4 × 10^−9^ mol gold in a volume of 1.78 mm^3^. Nuclear imaging is the clinical imaging modality with the highest sensitivity, as picomolar marker concentrations can be detected, due to the absence of intrinsic background [10,41]. MRI has a sensitivity for millimolar to micromolar marker concentrations [9,10], XFI therefore lies in-between. Lastly, image acquisition time in clinical modalities ranges from seconds (conventional X-ray and CT) to several minutes (MRI) and several minutes up to an hour (nuclear imaging) [7]. Our setup thus would lie in between MRI and nuclear imaging, depending on the size of the scanned region. Fan or cone beam XFCT is being researched as an alternative to achieve lower image acquisition times and add the benefit of volumetric imaging, but as a trade-off good sensitivity, dose load and image resolution are more difficult to achieve than when using a pencil beam [27,66]. The availability of markers and contrast agents is another advantage of XFI. Due to the specific fluorescence energies, similar to differently colored dyes, several markers could be traced simultaneously as opposed to PET tracers or MRI contrast agents. In comparison to that of the isotopes used in PET, the time-window for imaging is potentially longer because markers like GNPs do not decay (but may still undergo renal clearance). Furthermore, the growing use of nanoparticles in medicine will further reduce their cost and production effort.

In conclusion, XFI fits well into clinical imaging modalities for molecular imaging with regards to sensitivity, resolution, and image acquisition time. However, the biggest drawback in our setup is the high effort in technology as X-ray detectors and collimators at the size of the ones used in the simulations would be very costly. Thus, with current common detectors, poorer sensitivity is to be expected. Since X-ray sources like synchrotrons are less available than other imaging sources, our setup currently is impractical compared to already available imaging modalities.

### 4.5. Limitations of the Setup

A major drawback of our setup is the big hole at the side of the X-ray detector to move the voxel phantom inside. It was created to enable full mobility of the voxel phantom to be able to scan any voxel without its geometry intersecting with the detector. Likewise, in reality a 4π-detector would require holes to fit a patient in. However, this leads to a loss of detector area with high sensitivity. A workaround would be to simulate only parts of the voxel phantom so that the detector could be closed around them. Bearing clinical application in mind, however, a more flexible detector with an adjustable hole or the combination of several smaller ones might be a better solution. Still, for a possible future setup with multiple detectors, or flexible 4π-detectors, more simulations with 4π-detectors like ours can be helpful to determine their ideal layout.

A limitation in our work is that the abdominal scan was simulated only for three different incident beam angles and the thorax scan with only one incident beam angle. Also, those incident beam angles are not necessarily the ideal ones for their respective positions. To find a truly ideal setup, more simulations are needed for specific target tissues and scans. Nonetheless, this approximative approach was chosen, as possible clinical XFI still lies in the future and our work is a proof-of concept study. Still, our results are sufficient to highlight or confirm basic principles and challenges for clinical application in human-sized objects. Another limitation in our setup is that only the results for an incident beam of 10^9^ photons were examined. As described above, this decision was met to keep the dose in the beam volume below a level of 10 mGy. As photon flux directly affects scanning time and current synchrotrons are able to provide such a flux per second [14], it was reasonable to start here. Signal and dose increase linearly, whereas the significance only increases with the square root of the dose. Therefore, rather than increasing flux for a better signal yield, it is interesting for reducing scanning time. Based on a flux of 10^9^ photons/second, our scan of an abdominal slice would have taken 24 s for 5 mm steps limited to a region of interest, or 120 s for 1 mm steps. The time for moving the patient in between the steps and possibly comodal imaging for definition of the region of interest in advance has to be added in addition. Scanning of a 100 × 100 mm^2^ square would take approximately 3 h with a 1 mm^2^ resolution at this photon flux. However, synchrotron facilities like the P21 Beamline at PETRA III can deliver photons with a photon flux of 10^10^ to 10^12^ at an energy of 40 to 140 keV [67] (p. 20). A flux of up to 10^11^ photons/second would reduce that time by an order of 100 to slightly below 2 min.

Lastly, considering the simulated gold amounts in the abdominal scan, for a whole organ like the colon, the gold would most likely be distributed in a more random matter, with higher concentrations at certain points and lower at other ones, instead of being the same in every voxel.

## 5. Conclusions

The aim of our study is to present a numerical study on the feasibility of immune cell tracking with GNP-labeled T-cells and XFI in human-sized objects. We show that with the spatial filtering of 4π-spectra, when using a polarized, monoenergetic pencil beam with an energy close to the K-edge of gold, this is feasible with a minimum detection limit of down to 4.4 × 10^5^ cells in a volume of 1.78 mm^3^. Sensitivity and resolution ranging well within clinically available modalities for molecular imaging can be achieved. However, our simulations are based upon the use of costly X-ray detector technology and X-ray sources with currently limited accessibility. Therefore, the actual application of our results in a clinical setting depends on further technological advancements in those respective fields.

## Data Availability

The data presented in this study are available on reasonable request.
